# Comparison of 0.375% and 0.2% Ropivacaine for Postoperative Analgesia Using Ultrasound-Guided Pectoral Nerve Block in Patients Undergoing Breast Cancer Surgery: A Randomized Controlled Study

**DOI:** 10.7759/cureus.89599

**Published:** 2025-08-08

**Authors:** Deepak Aggarwal, Amit Singhal, Chetan Saraya, U K Valecha, Ashish Goel

**Affiliations:** 1 Department of Anaesthesiology, Kanti Devi Medical College and Hospital, Mathura, IND; 2 Liver Transplantation, B. L. Kapur (BLK) MAX Super Speciality Hospital, Delhi, IND; 3 Anaesthesiology, B. L. Kapur (BLK) MAX Super Speciality Hospital, Delhi, IND; 4 Anaesthesiology, Pain Management, B. L. Kapur (BLK) MAX Super Speciality Hospital, Delhi, IND; 5 Surgical Oncology, B. L. Kapur (BLK) MAX Super Speciality Hospital, Delhi, IND

**Keywords:** analgesia, breast cancer, modified radical mastectomy(mrm), pectoral nerve block(pecs), ropivacaine

## Abstract

Introduction: Breast cancer incidence has been rising in recent years, particularly among younger women, and it is now the leading cancer among Indian females. Acute postoperative pain is a significant concern, often deterring patients from surgery. Combining regional anesthesia with intravenous analgesics can improve postoperative outcomes. This study compares two concentrations of ropivacaine for managing postoperative pain in breast cancer surgeries.

Materials and Methods: Patients were randomized into two groups: Group 1 received 0.375% ropivacaine, and Group 2 received 0.2% ropivacaine via ultrasound-guided pectoral nerve block after anesthesia induction for breast surgeries. Baseline vitals were recorded. Intraoperative analgesia included fentanyl, IV paracetamol, and nerve block. Postoperative vitals and pain scores were monitored at regular intervals, with rescue analgesia provided through patient-controlled IV fentanyl. Statistical analysis used Chi-square or Fisher’s exact tests for nominal categorical data and Mann-Whitney U or Wilcoxon Rank Sum tests for non-normal continuous variables.

Results: Both groups were comparable in demographic data. Postoperative systolic blood pressure (SBP), diastolic blood pressure (DBP), and pulse rate (PR) measured at fixed intervals showed no significant differences between the groups. Pain scores on the numeric rating scale were also similar. While total 24-hour fentanyl consumption did not differ significantly, average fentanyl use was lower in Group 1.

Conclusion: This study found no significant difference in postoperative pain, antiemetic requirements, or patient satisfaction between 0.375% and 0.2% ropivacaine. While 0.2% ropivacaine is equally effective in terms of analgesia and patient satisfaction, 0.375% may be preferred in scenarios requiring prolonged sensory blockade. While 0.2% ropivacaine is equally effective in terms of analgesia and patient satisfaction, 0.375% may be preferred in scenarios requiring prolonged sensory blockade.

## Introduction

Pain, as defined by the International Association for the Study of Pain (IASP), is “an unpleasant sensory and emotional experience associated with actual or potential tissue damage or described in terms of such damage.” It is subjective, inherently unpleasant, and both sensory and emotional [1].

Effective postoperative pain management is individualized rather than standardized. Acute postoperative pain occurs within the first 7 days, while pain persisting beyond 3 months is classified as chronic. Both acute and chronic pain may originate from cutaneous, somatic, or visceral structures [2]. Pre-emptive analgesia is when ropivacaine is administered before surgery to reduce post-surgical pain by preventing central sensitization. Techniques include local anesthetic infiltration, nerve blocks, epidurals, subarachnoid blocks, intravenous (IV) analgesics, and anti-inflammatory drugs [3]. Multimodal analgesia combines opioid and non-opioid drugs targeting different mechanisms to minimize opioid use and side effects [4].

Strategies for pain control

Pre-emptive analgesia and multimodal analgesia leverage synergistic effects of diverse analgesic agents [5]. Breast cancer is the most common cancer among women globally, diagnosed increasingly at early stages due to improved detection and lifestyle factors. Pre-emptive, multimodal analgesia is recommended for breast cancer surgeries.

Surgery, often a critical component of treatment, necessitates effective strategies to alleviate associated anxiety and pain. Post-mastectomy, patients frequently experience acute nociceptive pain and chronic neuropathic pain [6]. Techniques like thoracic paravertebral and epidural blocks, or wound infiltration, have shown efficacy but are less suitable for day surgeries due to complications [7-14]. The pectoral nerve block (Pecs block) offers a safer alternative with fewer complications [15] and superior postoperative pain relief [16]. The pectoral nerve block is a relatively new technique designed to block nerves supplying the chest wall and axilla with minimal side effects. In 2013, Blanco introduced a variation of his original Pec I block by adding a local anesthetic injection between the serratus anterior and pectoralis minor muscles, known as the Pec II block. This modification extends analgesia to the axilla. Ropivacaine, preferred for its safety and rapid onset than bupivacaine, is used in our study [17-19].

The primary objective was to compare the duration of postoperative analgesia between 0.375% and 0.2% ropivacaine administered via ultrasound-guided pectoral nerve block in breast cancer surgery. Secondary objectives included assessing postoperative pain (numeric rating scale (NRS)), rescue antiemetic use, incidence of nausea and vomiting, patient satisfaction at 24 hours, and adverse events.

## Materials and methods

Research design

**Figure 1:**
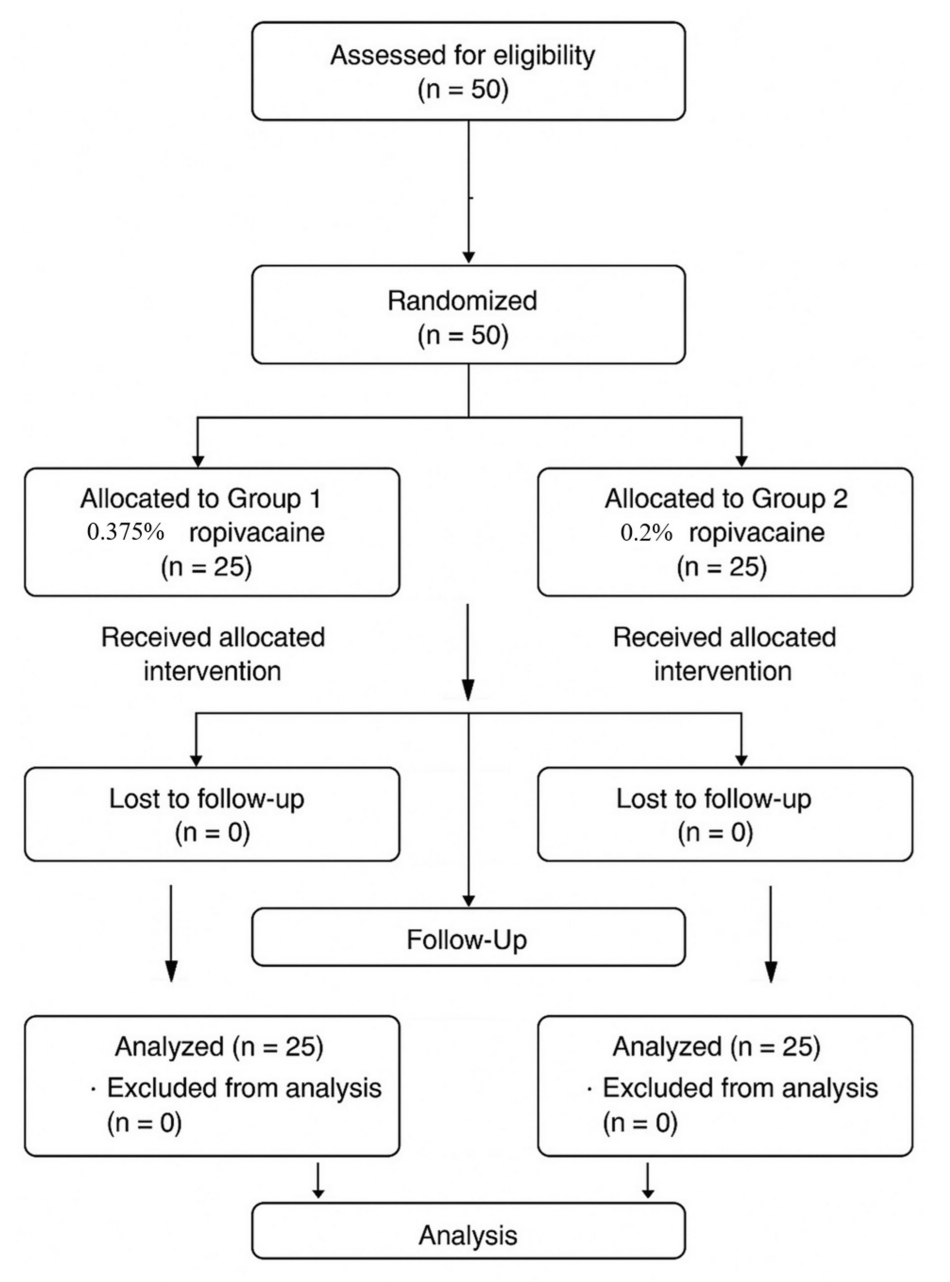
CONSORT diagram

This prospective, randomized, double-blind study aimed to assess the efficacy of two concentrations of ropivacaine (0.375% and 0.2%) for Pecs II and I blocks in patients undergoing breast cancer surgery. A total of 50 patients, aged 18-70 years, with ASA (American Society of Anesthesiologists) grade 1 or 2, were randomized into two groups. After receiving general anesthesia, an ultrasound-guided Pecs block was performed, and postoperative pain was managed using the NRS and patient-controlled IV fentanyl. The primary outcome, block duration, was defined as the time interval from the completion of the Pecs block administration to the patient’s first request for rescue analgesia (NRS score ≥ 4), which activated the patient-controlled fentanyl pump. Pain scores, vital signs, and fentanyl consumption were recorded at multiple time points, and overall pain relief was assessed 24 hours post-surgery. The block was administered by an experienced anesthesiologist who was blinded to the drug concentration, and intraoperative block efficacy was assessed by confirming surgical anesthesia prior to incision. Randomization in this study was performed using a computer-generated random sequence to ensure unbiased allocation. Patients meeting the inclusion criteria were assigned in a 1:1 ratio to either Group I (0.375% ropivacaine) or Group II (0.2% ropivacaine). Allocation concealment was maintained using sealed opaque envelopes opened just before performing the block. This approach minimized selection bias and preserved the double-blind nature of the trial. The sample size was calculated to detect a clinically significant difference of 90 minutes in the duration of postoperative analgesia between the two groups, assuming a standard deviation of 100 minutes, a significance level of 0.05, and 80% power. Using the formula for comparing two means, the required sample size was 20 patients per group. To account for possible dropouts, 25 patients were included in each group, making a total of 50 participants (Figure 1).

The primary objective of this study is to compare the duration of postoperative analgesia achieved with two different concentrations of ropivacaine (0.375% vs. 0.2%) administered via ultrasound-guided pectoral nerve block in patients undergoing breast cancer surgery. The secondary objectives are to evaluate and compare the two groups with respect to postoperative pain scores using the NRS, total requirement of rescue antiemetics within 24 hours, incidence of postoperative nausea and vomiting, patient satisfaction scores at 24 hours, and any adverse events associated with the interventions. The Ethical Committee of Dr. B. L. Kapur Memorial Hospital issued approval (Approval number: CTRI/2018/04/013105).

Inclusion and exclusion criteria

Table 1 shows the inclusion and exclusion criteria of the patients enrolled in this study.

**Table 1:** Inclusion and Exclusion Criteria ASA: American Society of Anesthesiologists

Inclusion Criteria	Exclusion Criteria
ASA grade 1 and 2 patients	Patients with respiratory or hepatic diseases
Aged between 18 and 70 years	Uncontrolled diabetes
Undergoing breast cancer surgery, including mastectomy or breast conservation with or without reconstruction.	Morbid obesity
Willingness to provide written informed consent.	Allergies to local anesthetics or ultrasound gel
Patients who can be randomized into either Group I (0.375% ropivacaine) or Group II (0.2% ropivacaine).	Chronic pain conditions
	Substance abuse
	Psychological disorders

Procedure and outcome assessment

Patients were divided into two groups: Group 1 received 0.375% ropivacaine, and Group 2 received 0.2% ropivacaine. Patients with respiratory or hepatic diseases, uncontrolled diabetes, morbid obesity, allergies to local anesthetics or ultrasound gel, chronic pain, substance abuse, or psychological disorders were excluded. Standard monitoring was applied in the operating room. The airway was secured using endotracheal intubation after induction of general anesthesia. This is evident because neuromuscular blockade was achieved with IV vecuronium (0.01 mg/kg) and later reversed with IV neostigmine and glycopyrrolate, which are used for reversal of muscle relaxants after intubation. Patients were extubated at the end of the procedure and then monitored in the post-anesthesia care unit (PACU). General anesthesia was induced with IV fentanyl (2 mcg/kg), propofol (2 mg/kg), and vecuronium (0.01 mg/kg), with maintenance on oxygen-air and sevoflurane (MAC 0.8-1.1). A post-induction ultrasound-guided Pecs block was performed on the surgical side by the same investigator. Using a linear array probe (6-18 MHz), the fascial planes of the pectoralis major, pectoralis minor, and serratus anterior muscles were identified at the 3rd rib. A 22-G Quincke spinal needle was inserted in-plane under ultrasound guidance. After confirming needle placement, 20 mL of the study drug was injected for the Pecs II block, followed by 10 mL for the Pecs I block as the needle was withdrawn. Intraoperative analgesia was managed with IV paracetamol (1 g) and additional fentanyl boluses (0.5 mcg/kg) if heart rate or blood pressure exceeded 20% of baseline. IV ondansetron (4 mg) was administered 30 minutes before surgery completion. Post-surgery, neuromuscular blockade was reversed with IV neostigmine (0.05 mg/kg) and glycopyrrolate (0.01 mg/kg). Patients were extubated and monitored in the PACU. Postoperative parameters (pain, vital signs including BP, HR, RR, and SpO2, and nausea/vomiting) were recorded at 1, 2, 4, 6, 12, and 24 hours. Pain was assessed using the NRS scale (0-10), with scores <3 indicating satisfactory relief. Rescue analgesia was provided using patient-controlled IV fentanyl (0.5 mcg/kg) with a 15-minute lockout. Total fentanyl consumption over 24 hours was recorded. After 24 hours, patients rated their overall pain relief on a 4-point scale: excellent, very good, satisfactory, or poor. Patient satisfaction was measured by asking patients to provide a self-reported satisfaction score 24 hours postoperatively. The satisfaction was rated on a numeric scale ranging from 1 to 3, where 1 indicated the lowest level of satisfaction, 2 represented moderate satisfaction, and 3 indicated the highest satisfaction. Patients were asked to reflect on their overall postoperative experience when providing their ratings.

Statistical analysis

Statistical analysis was performed using SPSS v17.0. Results were expressed as mean ± SD, median (min-max), or percentages. Student’s t-test and paired t-test were used for normally distributed variables, and Mann-Whitney U or Wilcoxon Rank Sum tests for non-normal data. Categorical variables were analyzed with Chi-square or Fisher’s exact tests. A p-value <0.05 was considered statistically significant.

## Results

Table 2 presents the baseline characteristics of patients in groups 1 and 2. Group 1 had a higher percentage of patients aged 31-40 years (20%) and 51-60 years (40%) compared to Group 2. Group 2 had more patients aged 41-50 years (36%) than Group 1 (4%). The mean age was 54.76 years for Group 1 and 52.64 years for Group 2, with no significant difference (P = 0.459). The mean weight was similar between groups (Group 1: 65.72 kg, Group 2: 64.32 kg, P = 0.64). Surgery duration was also similar (Group 1: 129 minutes, Group 2: 142.64 minutes, P = 0.792). ASA grade distribution showed no significant difference between groups (P = 0.248).

**Table 2:** Baseline characteristics of the patients in both groups *P<0.05

Parameter	Groups				P-Value*
	Group 1		Group 2		
Age Group	Frequency	%	Frequency	%	
31-40 yrs	5	20.00	2	8.00	0.037
41-50 yrs	1	4.00	9	36.00	
51-60 yrs	10	40.00	8	32.00	
>60 yrs	9	36.00	6	24.00	
Total	25	100	25	100	
Age	54.76 ± 10.51		52.64 ± 9.53		0.459
Weight	65.72 ± 9.35		64.32 ± 11.54		0.64
Duration of Surgery (mins)	129	142.64 ± 39.94	128	139.68 ± 38.85	0.792

Table 3 shows the distribution of ASA grades between the two groups, which shows that ASA grade 1 was observed in 32% of patients in Group 1 compared to 48% in Group 2, while ASA grade 2 was more frequent in Group 1 (68%) than in Group 2 (52%). The p-value for this comparison is 0.248, which is greater than 0.05, indicating that the difference in ASA grade distribution between the two groups is not statistically significant. Thus, both groups were comparable in terms of ASA physical status classification.

**Table 3:** ASA grade of the patients in both groups and their analysis ASA: American Society of Anesthesiologists

ASA Grade	Group				P-Value
	Group 1		Group 2		
	Frequency	%	Frequency	%	
1	8	32.00%	12	48.00%	0.248
2	17	68.00%	13	52.00%	
Total	25	100%	25	100%	

**Figure 2:**
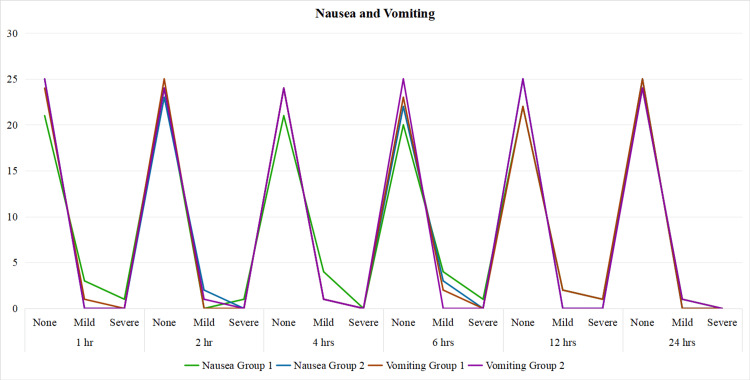
Nausea and vomiting in each group with respect to time

Figure 2 compares the incidence of nausea and vomiting across different time intervals (1, 2, 4, 6, 12, and 24 hours) in two groups. At all time points, the majority of patients in both groups reported no nausea or vomiting, while mild and severe cases were minimal. Trends for both nausea and vomiting are nearly identical between groups, with no significant differences observed, indicating comparable postoperative outcomes regarding these symptoms.

Numeric rating scale (NRS)

**Figure 3:**
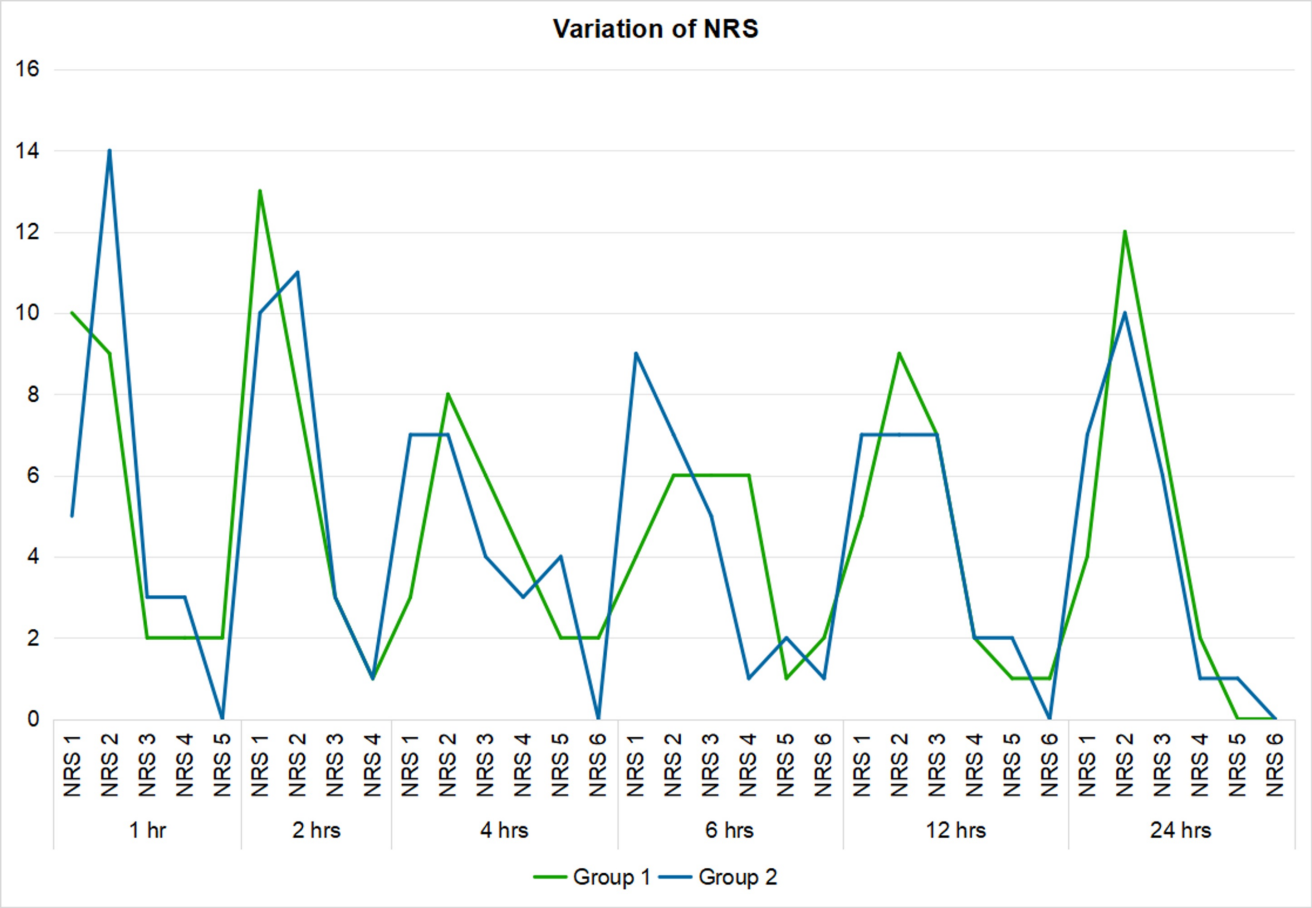
Variation of NRS in each group NRS: numeric rating scale

The postoperative pain score on NRS was not found to be statistically significant between the two groups (p>0.05). As can be seen in the table, the average NRS score is similar in both groups (Figure 3).

Table 4 shows the baseline characteristics of patients in the two groups. The analysis of categorical and continuous data between Group 1 and Group 2 reveals several differences. For the age group, Group 1 had 5 participants (20.00%) in the 31-40 years age group. In the present study, postoperative antiemetic requirement within 24 hours was comparable between the two groups. In Group 1 (0.375% ropivacaine), six patients (24%) required ondansetron, whereas in Group 2 (0.2% ropivacaine), three patients (12%) required it. The majority of patients in both groups did not require any antiemetic (76% in Group 1 vs. 88% in Group 2). Statistical analysis showed no significant difference between the groups (p = 0.463). These findings suggest that both concentrations of ropivacaine are similar with respect to postoperative nausea and vomiting control (Table 4).

**Table 4:** Postoperative assessment of the patients to be given antiemetics in both groups

Antiemetic 24 hr	Group				P-Value
	Group 1		Group 2		
	Frequency	%	Frequency	%	
Ondansetron	6	24.00	3	12.00	0.463
None	19	76.00	22	88.00	
Total	25	100	25	100	

Patients’ satisfaction

The distribution of patient satisfaction scores 24 hours post-treatment shows that in Group 1, 12 participants (48.00%) reported a score of 1, 9 participants (36.00%) reported a score of 2, and 4 participants (16.00%) reported a score of 3. In Group 2, 9 participants (36.00%) reported a score of 1, 12 participants (48.00%) reported a score of 2, and 4 participants (16.00%) reported a score of 3. The chi-square test for the association between the patient satisfaction score and group yielded a p-value of 0.651, indicating no statistically significant difference between the groups (Table 5).

**Table 5:** Patients’ satisfaction score (24 hours) degrees of freedom (df) = 2, and the effect size (Cramér’s V) = 0.132 (small effect)

Patient Satisfaction Score 24 hr	Group				P-Value
	Group 1		Group 2		
	Frequency	%	Frequency	%	
1	12	48.00	9	36.00	0.651
2	9	36.00	12	48.00	
3	4	16.00	4	16.00	
Total	25	100	25	100	

**Figure 4:**
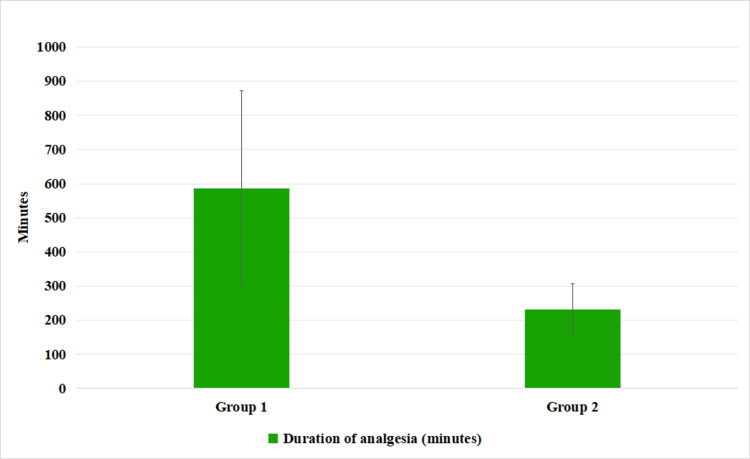
Duration of anesthesia as found in each group Duration is in minutes

In this study, Group 1 received 0.375% ropivacaine, and Group 2 received 0.2% ropivacaine. Figure 4 shows the sensory block duration. The mean duration of anesthesia for Group 1 was 586 minutes, with a standard deviation of 285.36 minutes, indicating a wide variation in anesthesia duration within this group. On the other hand, Group 2 had a mean duration of 231.2 minutes, with a standard deviation of 76.54 minutes, showing a more consistent and significantly shorter anesthesia duration compared to Group 1. This suggests that the higher concentration of ropivacaine in Group 1 (0.375%) resulted in a significantly longer anesthesia duration compared to the lower concentration (0.2%) in Group 2 (P=0.0269) (Figure 4).

## Discussion

This prospective double-blind study was conducted in the Department of Anaesthesiology, Dr. B. L. Kapur Memorial Hospital, New Delhi, to evaluate the efficacy of two concentrations of ropivacaine in Pecs block for postoperative analgesia in breast cancer surgery. Fifty ASA grade I and II patients aged 18-70 years were randomized into two groups of 25 each. Group I received 0.375% ropivacaine, and Group II received 0.2% ropivacaine after anesthesia induction.

Both groups were comparable in demographic variables, including age, weight, ASA grade, and surgery duration.

Postoperative pain is distressing and harmful, potentially leading to immobility, deep vein thrombosis, atelectasis, muscle wasting, and urinary retention [20]. Effective pain management improves surgical outcomes by reducing morbidity, hospitalization, and recovery time [21]. It also facilitates rehabilitation and may lower the risk of chronic pain [22]. Women undergoing breast surgery often experience chest wall, scar, or phantom pain (11-57%) and arm or shoulder pain (12-51%). Acute postoperative pain and analgesic requirements are strong predictors of persistent pain in the breast and ipsilateral arm [23]. Peripheral nerve blocks are used as part of a pre-emptive and multimodal analgesic technique to provide safe and effective postoperative pain management with minimal side effects.

Although the Pecs block was first described by Blanco in 2011 for pain relief in breast surgery, there were very few trials to validate its efficacy. The first study to validate its efficacy was done by Wahba et al. in 2014, in which they showed Pecs block was superior to thoracic paravertebral block, which was an established method of pain relief in breast surgery [16]. More recently, Kulhari et al. in 2016 again compared Pecs block with thoracic paravertebral block for patients undergoing breast surgery and came to the same conclusion that Pecs block is superior to thoracic paravertebral block in terms of duration of analgesia and opioid consumption [24].

Pecs block was compared against IV multimodal analgesia by Bashandy and Abbas in 2015, and it was again found to have better pain relief [25]. In 2016, Eldeen compared it with thoracic segmental spinal blockade, which was thought to have superior analgesia, but it was not proved in their study [26]. But the Pecs block was shown to have a significant prolongation of analgesic duration with a reduction in opioid consumption. Currently, there are no studies comparing different concentrations of ropivacaine in the Pecs block. However, Taha et al. have tried to find the minimum possible dose of ropivacaine to provide analgesia in femoral nerve block. They found that the minimal effective ropivacaine concentration was estimated to be 0.167% [27]. Similarly, Brodner et al. also found in their study that ropivacaine 0.2% and 0.3% provided effective analgesia in femoral nerve blocks when administered as a continuous infusion [28].

Yao et al. also tried to find the minimum possible analgesic dose in femoral-sciatic nerve block and concluded that 0.2% ropivacaine provided satisfactory postoperative analgesia, while preserving the ability of motion [29]. In our study, we tried to compare the efficacy of two concentrations of ropivacaine, i.e., 0.375% and 0.2% (Groups I & II), given in Pecs block I & II after induction of anesthesia for postoperative pain relief. The study also found that the NRS score was not significantly different between the two groups in 24 hours (p-value>0.05). As there were limited or no studies with different concentrations of local anesthetics in Pecs block, our study corroborated with other studies using the same local anesthetic drug in different concentrations in other peripheral nerve blocks.

Patients were asked to use the IV-patient-controlled analgesia (PCA) pump whenever they felt pain, i.e., rescue analgesia in boluses of 0.5 mcg/kg fentanyl. It was found that average total fentanyl consumption in micrograms was less in Group I (311.32± 166.63) than in Group II (411.56 ± 252.88), but it was not found to be statistically significant (p-value= 0.105). When other postoperative vital parameters, including pulse rate, blood pressure, and respiratory rate, were compared between the two groups, they were also found to be non-significant (p>0.05), which showed pain relief was good in both groups.

Overall, the incidence of nausea and vomiting was also equal in both groups (Group I with 0.375% and Group II with 0.2% ropivacaine) as shown by p-value >0.05. It was seen in a previous study done by Morioka et al., which showed that postoperative nausea and vomiting were equal in their study group with Pecs+GA and GA alone. No patient in either group in our study had any complications like bleeding, pneumothorax, or infection. Patients in both groups, when asked to rate their satisfaction level on a four-point scale, rated it equally in both groups (p-value 0.651). Thus showing that the patients in both groups had the same degree of pain relief after the surgery for the next 24 hr in the postoperative period.

However, this study had a limitation; ethically, we cannot wait for the patient to complain of pain and had to attach the PCA pump soon after the surgery. The PCA pump in use was not displaying the time to the first use of analgesics, and therefore, we could not calculate the time to rescue analgesia. Thus, we calculated the total dose of fentanyl consumed and the quality of pain relief obtained. Thus, more studies need to be done to validate the results of our research.

## Conclusions

This study found no significant differences in postoperative pain, antiemetic requirements, or patient satisfaction between 0.375% and 0.2% ropivacaine. However, the 0.375% concentration resulted in a significantly longer anesthesia duration compared to the 0.2% concentration. Both concentrations were similarly effective in pain management, with the choice depending on the desired duration of anesthesia. The choice between 0.375% and 0.2% ropivacaine depends on the procedure's requirements. If a longer duration of anesthesia is needed, such as for extended surgeries, 0.375% ropivacaine is preferable due to its longer-lasting effects. However, if a shorter anesthesia duration is sufficient, 0.2% ropivacaine may be better to minimize side effects while still providing adequate pain relief. This study compared the effects of two concentrations of ropivacaine (0.375% and 0.2%) on patients undergoing surgery. The baseline characteristics between the two groups were generally similar, with the exception of age distribution, where Group 1 had a higher percentage of patients aged 31-40 years and over 60 years. These differences, however, did not significantly impact the overall results of the study. In terms of postoperative pain scores, there was no statistically significant difference between the groups, suggesting that both concentrations provided similar levels of pain relief. Antiemetic requirements were also similar across the groups, with no significant difference in the need for ondansetron. Regarding patient satisfaction, the results were closely aligned in both groups, with no significant difference in overall satisfaction levels. However, a significant difference was observed in the duration of anesthesia, with Group 1 (0.375% ropivacaine) showing a notably longer duration compared to Group 2 (0.2% ropivacaine).

The small sample size reduces the statistical power and generalizability of the findings. There was an age distribution imbalance between groups, which may influence postoperative outcomes. Comorbidities were not uniformly controlled or analyzed, potentially introducing confounding factors.

This finding suggests that the higher concentration of ropivacaine in Group 1 provides a longer-lasting effect, which may be beneficial in certain clinical settings where prolonged anesthesia is desired. We found in our study that analgesia in terms of NRS score was equal in both groups. P-value showed a non-significant difference between the two groups. Total fentanyl consumption, although it was less in Group 1, it was not shown to be statistically significant. On this basis, we concluded that Pecs block I & II can be given with either concentration of ropivacaine for postoperative pain relief in breast cancer surgery.

Therefore, it is recommended that the lower concentration of 0.2% should be used for postoperative pain relief in pectoral nerve block, as a higher concentration of 0.375% has no additional analgesic benefit.
